# The link between mental rotation ability and basic numerical representations

**DOI:** 10.1016/j.actpsy.2013.05.009

**Published:** 2013-10

**Authors:** Jacqueline M. Thompson, Hans-Christoph Nuerk, Korbinian Moeller, Roi Cohen Kadosh

**Affiliations:** a Department of Experimental Psychology, University of Oxford, South Parks Road, OX1 3UD Oxford, UK; b Department of Psychology, Eberhard Karls University, Schleichstrasse 4, 72076 Tuebingen, Germany; c Knowledge Media Research Center, IWM-KMRC, Schleichstrasse 6, 72076 Tuebingen, Germany

**Keywords:** Mental rotation, Numerical representation, Compatibility effect, Numerical cognition, Number line, Spatial abilities

## Abstract

Mental rotation and number representation have both been studied widely, but although mental rotation has been linked to higher-level mathematical skills, to date it has not been shown whether mental rotation ability is linked to the most basic mental representation and processing of numbers. To investigate the possible connection between mental rotation abilities and numerical representation, 43 participants completed four tasks: 1) a standard pen-and-paper mental rotation task; 2) a multi-digit number magnitude comparison task assessing the compatibility effect, which indicates separate processing of decade and unit digits; 3) a number-line mapping task, which measures precision of number magnitude representation; and 4) a random number generation task, which yields measures both of executive control and of spatial number representations. Results show that mental rotation ability correlated significantly with both size of the compatibility effect and with number mapping accuracy, but not with any measures from the random number generation task. Together, these results suggest that higher mental rotation abilities are linked to more developed number representation, and also provide further evidence for the connection between spatial and numerical abilities.

## Highlights

• We show a link between mental rotation ability (MRA) and numerical representation.
• MRA correlated with a measure of holding concurrent multiple number representations.
• MRA correlated with a measure of number representation precision (number line task).
• MRA did not correlate with an executive control task (random number generation).
• These findings strengthen the link observed between spatial and numerical abilities.

## 1 Introduction

A strong connection has long been noted between mathematical and spatial cognitive abilities. Studies of developmental, individual, and sex differences among cognitive skills have consistently shown that spatial aptitude and mathematical aptitude tend to align (Geary, Saults, Liu, & Hoard, 2000; Reuhkala, 2001). However, it is unclear whether this connection exists solely with high-level mathematical abilities or if it is founded upon a deeper overlap between spatial abilities and basic numerical cognition. Most of the current evidence has established connections between spatial abilities and high-level numerical abilities, such as mathematical abilities (e.g., Dumontheil & Klingberg, 2012). However, it is possible that such connections are based on a more fundamental link between spatial abilities and basic numerical abilities that serve as the building block for high level numerical abilities (Butterworth, 2010). Despite the common intuition that numbers are represented purely abstractly (for a review see Cohen Kadosh & Walsh, 2009), numerical cognition has been shown to incorporate a vigorous spatial component; for instance, spatial influences have been shown on numerical tasks such as number interval bisection, parity judgment, and numerical value comparison, whereas irrelevant but automatically-processed numbers have been shown to influence spatial tasks such as attentional cueing and physical line bisection (e.g., de Hevia, Vallar, & Girelli, 2008; Vallar & Girelli, 2009). Space is a powerful conceptual framework for learning number properties of ordering and magnitude, as illustrated in the embodied cognition account of Lakoff and Nunez (2000), and as evidenced in the widespread use of spatial number lines in early mathematics education (Ernest, 1985). Additionally, lesion and imaging studies have implicated common areas in the parietal cortex for both spatial (e.g., physical line bisection, spatial attention and orientation) and numerical (e.g., number comparison, numerosity and magnitude judgment) abilities, suggesting that they may recruit shared neural circuits (for reviews see Cantlon, Platt, & Brannon, 2009; Cohen Kadosh, Lammertyn, & Izard, 2008; Hubbard, Piazza, Pinel, & Dehaene, 2005; Walsh, 2003). It follows, then, that spatial and numerical cognitive abilities may indeed be closely linked in the nature of their representation.

### 1.1 Mental rotation

Mental rotation has proven to be a robust and popular measure of spatial ability, particularly for spatial representation and mental manipulation of objects (Borst, Kievit, Thompson, & Kosslyn, 2011; Poltrock & Brown, 1984). Mental rotation is a computationally complex spatial process, with performance varying widely across individuals irrespective of other intelligence measures (Borst et al., 2011; Johnson & Bouchard, 2005; Shepard & Metzler, 1971). Opinions have varied as to how mental rotation fits within the subset of observable spatial skills, and how these skills ought to be grouped or classified in terms of mental processes (for instance, see Voyer, Voyer, & Bryden, 1995). Despite this disagreement, mental rotation has nonetheless been shown to correlate with other tests of spatial abilities, such as mental paper-folding tasks, space relations tests, and spatial working memory (Just & Carpenter, 1985; Kaufman, 2007; Reuhkala, 2001), suggesting that it may predict, at least to some degree, more general spatial skills of a participant. This extrapolation to other spatial abilities may occur in the form of spatial object mapping; according to converging neuroimaging evidence, mental rotation appears to recruit posterior parietal areas implicated in spatially-mapped analog representations (for a review see Zacks, 2008). Both behavioral and imaging evidence suggest that mental rotation tasks evoke visuospatial representations corresponding to object rotation as seen in the physical world, through graded transformational processes working upon analog object representations. For instance, Shepard and Metzler (1971) demonstrated that response latencies in a mental rotation task varied as a linear function of rotational angle between the target and comparison object. Furthermore, several fMRI studies have since found neural correlates for this behavioral effect, showing that bilateral parietal lobe activation increases as a function of rotational angle in mental rotation of objects, both when visually presented (Carpenter, Just, Keller, Eddy, & Thulborn, 1999; Gogos et al., 2010) and when retrieved from memory (Just, Carpenter, Maguire, Diwadkar, & McMains, 2001).

### 1.2 Number representation

Like mental rotation, basic number representation has been widely investigated. By “number representation” or “numerical representation” we simply mean the mental organization and framework within which information about the cognitive concept of numbers is stored. Thus number representation is the most basic level of numerical cognition upon which all other (more complex) numerical and mathematical thinking builds. While this basic number representation must ultimately have a neuronal basis, it is important to remember that representation and neuronal organization are not necessarily the same thing, and that a particular proposed system of number representation could have many possible neuronal manifestations. In this article it is equally important to distinguish what we will refer to as “number processing”: the nature of processing necessarily relies upon the underlying representation of the concepts and percepts being acted upon, but it is not a synonym for representation. Rather, it refers to the *act of engaging* mental representations, in order to use this numerical information for number-related tasks or other cognitive processes. Therefore, since number representation is not accessible by any means other than number processing, observation of number processing is the only way to infer aspects of the underlying representation.

It is similarly worthwhile to explain here the distinction between, on the one hand, *numerical* skills, abilities, or processing, and on the other hand, *mathematical* skills, abilities, or processing. The relationship between these two concepts is a nested one; numerical skill is only one component part of mathematical skill. In this model, numerical skills necessarily rely heavily—perhaps primarily—upon numerical representation, with few other basic sub-processes mediating their outcomes, such as visual recognition of numerals. On the other hand, mathematical skills additionally rely upon (and therefore can be amplified or attenuated by) a greater number, degree, and complexity of sub-skills and sub-processes, such as logical inference, memorization of calculation procedures, working memory, etc. For instance, factors such as working memory have been shown to predict later mathematical performance in a longitudinal developmental study (Moeller, Pixner, Zuber, Kaufmann, & Nuerk, 2011). Thus, to investigate numerical representation we utilized tasks that engage numerical, rather than mathematical, skills, as mathematical measures may be affected by a multitude of these non-numerical factors.

Details of number representation have been inferred from several types of tasks, including number line mapping and numerical comparisons. Numerical comparison tasks ask participants to indicate which of the two numbers is larger in magnitude (or sometimes, smaller in magnitude). This requires participants to access mental representations of the numerical magnitude of each number, and to perform comparative processes on these representations. Such tasks show several reliable behavioral effects, each shedding light on the inner workings of number processing and underlying representation. One of the effects, the unit-decade compatibility effect, arises from the decimal place-value structure of symbolic Arabic numbers. It provides evidence for decomposed processing of multi-digit numbers, thereby challenging a previous suggestion that numbers are represented by a single holistic representation, i.e., as an integrated entity which does not retain place-value information (Dehaene, Dupoux, & Mehler, 1990). The compatibility effect reflects a performance cost for trials in which the magnitude decision between unit digits of the two numbers is incompatible with (that is, opposite to) the magnitude decision between the decade digits (e.g., for a ‘compatible’ trial, such as 42 vs. 57, 4 < 5 and 2 < 7; but for an ‘incompatible’ trial, such as 37 vs. 52, 3 < 5 but 7 > 2; Nuerk, Weger, & Willmes, 2001). The performance cost for incompatible trials suggests that the unit digits of two-digit numbers are automatically processed, even when they are irrelevant to the task.

Further evidence using eye-tracking supports this interpretation, indicating that participants showed more eye fixations on unit digits than decade digits, and especially so for incompatible trials (Moeller, Fischer, Nuerk, & Willmes, 2009). This pattern has been interpreted as reflecting the need to inhibit magnitudes of unit digits for incompatible trials only, as the (irrelevant) unit comparison interferes with the decade and overall comparison; therefore the data are most consistent with a model in which both digits are processed separately (see Moeller, Fischer, et al., 2009, for a detailed version of that argument including hypothetical eye fixation patterns for various models and conditions). Such separate processing requires the activation of multiple representations, at least one for each digit. Thus the compatibility effect can serve as a quantifiable measure indicating the robustness of simultaneous processing of multiple (i.e., decomposed-digit) numerical representations (for a review, see Nuerk, Moeller, Klein, Willmes, & Fischer, 2011). This would seem to indicate that larger compatibility effects would accompany a more complex, advanced system of number representation; indeed, developmental studies of the compatibility effect have shown it to increase with age and numerical experience (Mann, Moeller, Pixner, Kaufmann, & Nuerk, 2011) and to predict later arithmetic ability (Moeller et al., 2011).

Another type of numerical task, the number line mapping task (also termed number line estimation task), has been widely utilized in the last decade as a measure of internal spatial representations of number in both children and adults (Cohen Kadosh, Soskic, Iuculano, Kanai, & Walsh, 2010; Karolis, Iuculano, & Butterworth, 2011; Siegler & Opfer, 2003). In its commonly used number-to-space version, the paradigm typically presents participants with a horizontal line segment labeled with a numerical value at either end (usually 0 at the left, and 10, 100, or 1000 at the right), and asks them to mark the place at which a target number should be located on the line. The deviation of this mark from the true position of the number on a linear equidistance line is assessed, and both absolute deviations as well as the form of these deviations are modeled to explore the possible underlying magnitude representation (see Moeller, Pixner, Kaufmann, & Nuerk, 2009; Siegler & Opfer, 2003; Slusser, Santiago, & Barth, 2012 for different suggestions). Developmental studies show that children's mean absolute error percentages on number line tasks drop below a threshold of 10% by age 8 for numbers 0–100, and by age 10 for numbers 0–1000, and that performance in this task predicts later arithmetic learning (Booth & Siegler, 2006, 2008). In addition, numerate adults are so accomplished at this number-mapping task that their results often show strong ceiling effects (Karolis et al., 2011; Siegler & Opfer, 2003). However, these can be ameliorated by the task format, for instance by varying the endpoints of the line to values other than 0 or exponents of 10. This reduces the efficacy of algorithms that can partition lines according to the overlearned concept of decimal structure, rather than relying upon the internal spatial number representation (Karolis et al., 2011).

In a less explicit fashion, random number generation (RNG) tasks have also been shown to reveal elements of fundamental number representation, through the form of inherent spatial biases. Specifically, when asked to produce a string of numbers between a specified minimum and maximum, in as random an order as possible, participants show a small but reliable bias to produce a greater proportion of relatively small numbers (Bachmann, Fischer, Landolt, & Brugger, 2010; Loetscher & Brugger, 2007). Loetscher, Schwarz, Schubiger, and Brugger (2008) found that this small-number bias (SNB) is especially pronounced with the experimental manipulations of asking participants to turn their heads to the left, and/or to imagine the numbers on a (left-to-right) ruler. This suggests that the basic representation of numbers, as accessed in the RNG task, incorporates a highly spatial aspect, and that this task may reflect the strength with which individuals represent numbers spatially (left-to-right). In addition to SNB, further reflections on number–space interaction in the RNG task can also be gleaned through additional, more complex measures of how the changing pattern of response choices “moves” along the number line (Loetscher & Brugger, 2009).

As its name suggests, the RNG task also provides various measures of response randomness, in which the sequence of random numbers produced by the participant is analyzed for similarity to actual random (or pseudo-random) sequences. In contrast to the other numerical tasks, or the spatial RNG indices, measures of randomness in RNG do not require and are not thought to directly reflect upon any explicit numerical magnitude representations (Brugger, 1997). Rather than relying on numerical skills, success at this measure is interpreted to rely mainly upon more general executive function, namely the ability to suppress response preferences created by one's own previous output (Brugger, 1997; Peters, Giesbrecht, Jelicic, & Merckelbach, 2007; Terhune & Brugger, 2011).

### 1.3 The present study

Surprisingly, despite extensive research on both mental rotation (for reviews see: Peters & Battista, 2008; Zacks, 2008) and basic number processing (for reviews see: Cohen Kadosh et al., 2008; Cohen Kadosh & Walsh, 2009), there is a lack of previous research satisfactorily explaining how these two cognitive faculties may relate to one another. Given the observed anatomical (Cantlon et al., 2009; Cohen Kadosh et al., 2008; Hubbard et al., 2005; Walsh, 2003) and behavioral (de Hevia et al., 2008; Dumontheil & Klingberg, 2012; Vallar & Girelli, 2009) overlap between numerical and spatial processing, it seems likely that spatial and numerical cognition may share common neurocognitive mechanisms, or did so at an earlier developmental (Cohen Kadosh, 2011; de Hevia, Girelli, & Macchi Cassia, 2012; Johnson, 2011), or evolutionary stage (Anderson, 2007, 2010; Dehaene & Cohen, 2007). Therefore, the present study set out to investigate whether the well-studied spatial faculty of mental rotation may indeed show cognitive links to basic numerical representation. As spatial abilities almost certainly evolutionarily predate cognition of symbolic numbers, one likely possibility driving such proposed links is that mental rotation and basic numerical skills both rely upon—or have developmentally derived from—shared spatial representation mechanisms. If this is the case, we would expect to see correlations between individual differences in spatial and numerical tasks which recruit such mechanisms.

Moreover, observing the types of measures that correlate should yield clues as to the more specific nature of these general posited mechanisms. To analyze these clues, however, we must also take into account the nature of the numerical representation. Namely, numerical representation can be characterized separately in terms of quality, or precision of representations, and in terms of quantity, or the feasibility of holding multiple simultaneous representations. Highly precise—that is, highly detailed and accurate—spatial representations should offer an advantage in mental rotation tasks, by helping to choose between response alternatives which may differ only in small details of feature orientation or length. They should also enhance accuracy in number-mapping tasks, because when spatial representations are more accurate, then individuals should be able to produce more accurate mappings in physical space. Therefore, spatial deviations should be less pronounced and consequently performance in the number line mapping tasks, which is measured by such spatial deviations, should be better. However, highly precise spatial representations should not show any discernible effect on tasks which compare the differential weight of holistic versus decomposed representation of multi-digit numbers, since both of these types of representations should similarly benefit from extra precision (by improving accuracy and processing time). Lastly, highly precise spatial representation of numbers might be expected to result in more pronounced spatial measures on RNG tasks, such as stronger small number bias or more complex patterns of response choice “moving” along the mental number line.

Ability to hold multiple simultaneous spatial representations should help in mental rotation tasks, as the task requires participants to represent and compare spatial features of two or more objects. Although strategies at this task may differ (Butler et al., 2006; Heil & Jansen-Osmann, 2008), both holistic object rotation and piecemeal feature-by-feature comparison require the comparison of two or more spatial representations; therefore, ability to hold multiple simultaneous spatial representations ought to aid in performance at this task. Additionally, this ability should be associated with a higher compatibility effect, as the effect arises from presence of multiple simultaneous (i.e. decomposed) representations of spatially separated digits in multi-digit numbers. It is unlikely, however, to affect accuracy of number line mapping, as the task is designed to reflect a single, holistic representation of numerical magnitude. Similarly, the RNG task does not seem likely to recruit multiple spatial representations of number, as it utilizes only single digits which are processed unidimensionally across time.

Therefore, if some individuals can create more precise and multiple spatial representations, this would predict a confluence of 1) better performance at spatial tasks, such as mental rotation, that require holding multiple 3-D representations with minimally different spatial features, 2) worse performance on tasks that evoke processing of irrelevant extraneous numerical representations (i.e., a larger compatibility effect), 3) better performance at spatial-numerical tasks, such as number line mapping, and 4) more pronounced effects of the spatial measures of RNG tasks.

## 2 Methods

### 2.1 Participants

Forty-three university students (mean age 21.26 years, *SD* = 2.94, 34 female, 5 left-handed) participated in the following four tasks: 1) the redrawn Vandenberg & Kuse pen-and-paper Mental Rotation Test (Peters et al., 1995); 2) a computerized numerical comparison task (Nuerk et al., 2001); 3) a computerized number-line mapping task (Cohen Kadosh et al., 2010); and 4) a verbal random-number-generation task (Loetscher & Brugger, 2007). All participants had normal or corrected-to-normal vision. Participants completed the four tasks in a single experimental sitting with order of tasks balanced across participants by a Latin square design.

### 2.2 Mental rotation task

Participants were administered a pen-and-paper version of the Peters et al. (1995) redrawing of the Vandenberg & Kuse Mental Rotation Test, consisting of 24 questions each showing several 2-D drawings of a 3-D block object, and asking participants to choose which two out of the four drawings on the right could be rotated to match the target drawing on the left. Participants were given 3 min to finish each 12-question section. According to the preferred scoring method suggested by Peters et al. (1995), to minimize effects of guessing, a point was awarded for a question only if both responses for that question were correct. Thus possible scores ranged from 0, which indicates poor mental rotation abilities, to 24, which indicates high mental rotation abilities.

### 2.3 Numerical comparison task

#### 2.3.1 Stimuli

Stimuli were displayed on a 19-inch flat-screen Dell monitor in Arial size 50 font, at a distance of ~ 55 cm. The two numbers in each trial were arranged horizontally, one on the left and one on the right, each at 2.1° visual angle from center.

#### 2.3.2 Procedure

Participants completed 480 trials of a dichotomous forced-choice, speeded numerical comparison task in which they indicated which of a pair of two-digit numbers was numerically larger. Participants responded by key-press with right or left index finger on the side of the chosen number (P or Q on QWERTY keyboard). They were instructed to respond as quickly and as accurately as possible. Each trial began with a fixation cross presented in the middle of the screen for 300 ms, followed by the number pair 300 ms after the offset of the fixation cross. The number pair remained on the screen until either P or Q was pressed, up to a maximum of 5000 ms. A new trial began 200 ms after the participant's response, and a participant-terminated break occurred after each 120 trials.

#### 2.3.3 Design

Number pairs were adapted from Moeller, Fischer, Nuerk, and Willmes (2009) and were balanced such that both relevant groups of stimuli (i.e. compatible versus incompatible trials) yielded statistically similar measures of multiple factors including overall numerical distance, unit distance, decade distance, problem size, correct hand response, and within-pair direction of numerical ordering. Half of the stimuli were within-decade comparisons (e.g., 51 vs. 58) to ensure that the unit digits were equally as relevant as decade digits in making the numerical comparisons throughout the task; however, as is typical for this paradigm, only between-decade number pairs were later analyzed. Between-decade pairs were classified as compatible or incompatible. The compatible condition comprised number pairs for which separate comparisons of tens and units yielded the same decision (e.g., 23 vs. 46; 2 < 4 AND 3 < 6); in contrast, the incompatible condition comprised pairs for which these single digit-comparisons differ in their direction (e.g., 26 vs. 53; 2 < 4 BUT 6 > 3). The frequency of compatible condition and incompatible condition was equal.

### 2.4 Number line mapping task

#### 2.4.1 Stimuli

Each trial presented a blue horizontal axis stretching from left to right, centered vertically on a black background screen and labeled with an anchor number on each end, always with the numerically smaller anchor on the left. To avoid center- or side-bias, target numbers were displayed in both upper-left and upper-right corners above the numerical anchors (e.g., −1000 and 1000). To differentiate between target and anchor numbers, target numbers appeared in yellow.

#### 2.4.2 Procedure

Participants completed 60 trials of the task, in which they indicated by mouse click where on the given number line a given target number should be mapped. Each trial was presented immediately following the mouse click of the previous. Trials were not restricted in terms of time, but participants were instructed to reply as accurately as possible while still going at a “reasonably fast pace,” which based on observation was rarely more than 20 s (1.7% of trials) and never more than 40 s.

#### 2.4.3 Design

Half the trials displayed a fixed range from −1000 to 1000. The other half of trials had variable axis ranges (e.g., 20 to 85, −100 to 400).

### 2.5 Random-number-generation task

#### 2.5.1 Stimuli

Participants heard an auditory stimulus of electronic beeps, with one beep per second, created with the software *Audacity* and played on a MacBook Pro laptop speaker.

#### 2.5.2 Procedure

The task asked participants to verbally produce a string of random numbers between 1 and 6 inclusive, to the rhythm of an auditory stimulus playing beats at a rate of 1 Hz. Participants were exhorted to give numbers as random as possible, as if rolling a die. The experimenter notated the produced numbers by hand to collect a total of at least 66 valid responses. Invalid responses (e.g., “0” or “7”) were infrequent (0.15% of responses) and were excluded from the analysis. Both invalid responses and skipped beats were tallied and recorded as separate variables.

#### 2.5.3 Design

The number strings produced were examined according to the methods of Towse and Neil (1998) for their similarity to true randomly generated number sequences, by analyzing individual response frequency, first-order differences, repetition distance, and response phase.

## 3 Results

### 3.1 Mental rotation task

Results were scored according to Peters et al. (1995), as described in Section 2.2, resulting in a score between 0 and 24. Scores were normally distributed, and the mean score across all participants was 11.58 (*SD*: 4.82), in line with previous findings of means around 11 (e.g., for comparison to a large student sample see Peters et al., 1995).

### 3.2 Numerical comparison task

**Fig. 1:**
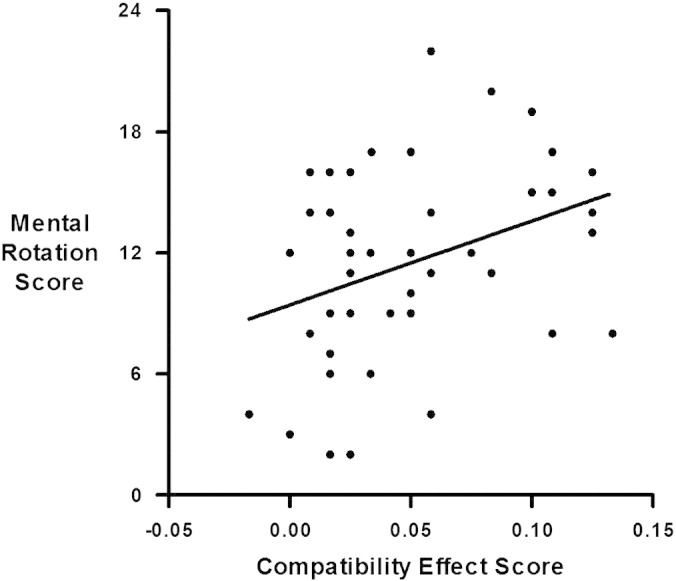
Mental rotation score correlates positively with size of the compatibility effect as measured by accuracy. The higher the mental rotation score the higher the compatibility effect, a measure which indicates higher degree of decomposed (separate-digit) processing of multi-digit numbers.

Mean reaction times (RTs) were calculated on correct trials only (mean error rate: 4.27%). Error rates were arcsine transformed before analysis to ensure that they approximated a normal distribution. Reproducing earlier findings (Nuerk, Weger, & Willmes, 2004; Nuerk et al., 2001), we found a significant main effect of compatibility (incompatible versus compatible trials) for both RTs (paired *t*(42) =17.26, *p* < .001) and accuracy (paired *t*(42) = 8.14, *p* < .001). We computed the compatibility effect for each individual participant by subtracting the mean score of all compatible trials from that of all incompatible trials. The correlation of mental rotation score (MRS) and compatibility effect using RTs was not significant (*r* = .04, *p* = .81). However, the correlation of MRS and compatibility effect measured by accuracy was significant (*r* = .4, *p* < .01, Fig. 1). Furthermore, this correlation was still significant even when controlling for RT in a partial correlation (*r* = .4, *p* < .01), indicating that it was not due to a speed–accuracy trade-off.

### 3.3 Number line mapping task

**Fig. 2:**
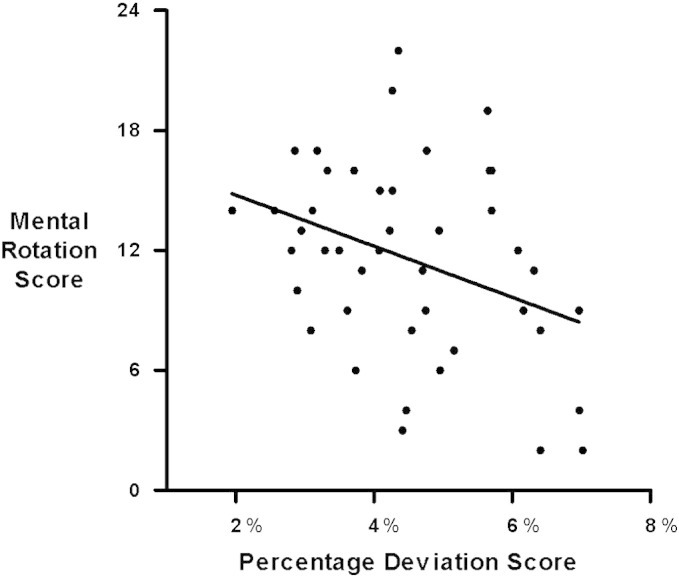
Mental rotation ability is negatively correlated with percentage of error (percentage deviation score) in mapping numbers on a number line.

A percentage deviation score was calculated for each participant by dividing the absolute deviation of their response from target value by the length of the numerical span represented on the axis (|subjective mapping − objective mapping| / numerical length of the axis; as in Booth & Siegler, 2006). This ensured that all trials were weighed equally in the analysis, regardless of the numerical axis length. A Pearson correlation revealed that the percentage deviation score was significantly negatively correlated with MRS (*r* = − 0.35, *p* < .05, Fig. 2), indicating that a more accurate number mapping (lower deviation) tended to co-occur with higher MRS. Average RT was 6.87 s (*SD*: 2.64). There was no correlation between RT and MRS (*r* = .05, *p* = .77). To ensure that the correlation between MRS and percentage deviation score did not arise from a speed–accuracy trade-off, we confirmed that the same relationship of percentage deviation score to MRS was still noted in a partial correlation controlling for RT (*r* = − .4, *p* < .01).^1^

Location-marking tasks, such as line bisection, often show an effect referred to as pseudoneglect: namely, participants tend to point to the left of the actual target location, analogous to behavior observed in perceptual hemispheric neglect (for a meta-analysis and review, see Jewell & McCourt, 2000). To ensure that our deviation score did not simply reflect effects of pseudoneglect rather than actual overall accuracy, for each participant we also calculated mean percentage deviation scores from the non-absolute (raw percentage) values of deviation, which includes information about direction (negative values for leftward deviation, and positive values for rightward deviation.) Analysis of these data revealed a small but significant population pseudoneglect, with mean leftward deviation of 0.72% of the given line (one-sample *t*(42) = 4.27, *p* < .001). However, as this measure was uncorrelated with MRS (*r* = .007, *p* = .97), we conclude that the precision of number-mapping, rather than systematic leftward pseudoneglect, is linked to mental rotation ability.

Although accuracy on the number-mapping task and size of compatibility effect were both significantly correlated with MRS, these two measures were not significantly correlated to each other (*r* = .13, *p* = .40).

### 3.4 Random number generation task

For each participant's set of 66 responses, several separate measures of random number generation were calculated, using the RgCalc program created by Towse and Neil (1998). We analyzed three measures of randomness. Redundancy of responses (R score) measures how often participants repeat each response choice; a score of 0% represents perfect equality of frequency among all choices, and 100% represents complete repetition of one choice. The RNG index measures the distribution of digrams, or pairs of response choices; a score of 0 represents perfect equality of digram frequency among all possible 36 combinations, and a score of 1 represents complete repetition of one choice. The RNG2 index is similar to the RNG index, but calculates it instead with pairs of digrams. None of these three measures correlated significantly with MRS: (R score: *r* = − .12, *p* = .44; RNG index: *r* = − .02, *p* = .92; RNG2 index: *r* = − .12, *p* = .42).

Additionally, we analyzed three spatial measures of RNG performance. Small number bias (SNB), as mentioned in Section 1.2, reflects a bias to spontaneously produce more small numbers than large numbers. SNB was thus calculated by finding the numerical difference between number of relatively “small” responses (1, 2, or 3) and number of “large” responses (4, 5, or 6). First-order differences (FODs) refer to the mathematical difference between each randomly generated number and the previous response. Thus positive FODs indicate a rightward direction of responses along the mental number line (e.g., 3 followed by 5 gives an FOD of + 2) and negative FODs indicate a leftward shift (e.g., 6 followed by 1 gives an FOD of − 5). Similarly to the SNB index, a measure of FOD differential was calculated by taking the numerical difference between number of “rightward” (positive) FODs and number of “leftward” (negative) FODs. Lastly, the turning point index (TPI) measures the number of changes in direction (positive or negative) of FODs compared to the expected number of such changes. For instance, the sequence 1-2-5-3-1 would show one such change, at response “5”: from ascending (positive) to descending (negative) sequences. Thus, the TPI measures how relatively often an individual “changes direction” along the mental number line within their string of responses. Neither SNB nor FOD measures were significantly different from 0 across the population (SNB: one-sample *t*(42) = 1.48, *p* = .15; FODs: one-sample *t*(42) = 1.40, *p* = .17). Mean TPI was 93.86, significantly less than the standard of 100 (one-sample *t*(42) = 3.73, *p* < .01), indicating that participants switched between ascending and descending sequences slightly less than expected by chance. However, none of these three measures correlated significantly with MRS: (SNB: *r* = − .04, *p* = .80; FODs: *r* = − .20, *p* = .21; TPI: *r* = − .002, *p* = .99).

## 4 Discussion

The current study endeavored to examine the link between mental rotation abilities and basic numerical representations, specifically investigating the hypothesis that both recruit detailed spatial representation abilities. To do so, we assessed both 1) mental rotation abilities and 2) basic numerical skills that tap the underlying numerical representations of the mental number line and the place-value structure of the Arabic number system (Loetscher & Brugger, 2007; Nuerk et al., 2001; Siegler & Opfer, 2003). We found a correlation between mental rotation performance (which relies heavily upon spatial representation and processing) and both 1) size of the compatibility effect, a measure which indicates the tendency to represent two-digit numbers by using multiple representations for tens and units, rather than a single holistic representation (Moeller, Fischer, Nuerk, & Willmes, 2009; Nuerk et al., 2001); and 2) accuracy of number line mapping, which is suggested to reflect precision of spatial number representation (Karolis et al., 2011; Siegler & Opfer, 2003). In contrast, performance in the mental rotation task did not correlate with measures of either randomness or spatial aspects in the random number generation task.

Although these null findings regarding the RNG measures ran counter to our original prediction, they are nevertheless easily reconciled with that initial hypothesis. Whereas the numerical comparison task and number line mapping task explicitly tap number magnitude representations, elicitation of number magnitude in the RNG task is purely implicit, meaning that the relationship of these effects to spatial representation may have been too subtle to measure in a correlation with mental rotation skills. This is consistent with previous research from Priftis, Zorzi, Meneghello, Marenzi, and Umilta (2006) showing that spatial–numerical impairments in neglect are only found in tasks drawing on explicit number knowledge (e.g., mental number line bisection) but not implicit number knowledge (e.g., spatial–numerical association of response codes, or SNARC). Therefore, together, the foremost and most parsimonious interpretation of our aggregate data is that a greater ability to process multiple, precise spatial representations subserves both superior mental rotation ability and more advanced (i.e. more closely approximating the adult end of the developmental trajectory) number representations. The observation that size of compatibility effect and accuracy of number-line mapping both correlated with mental rotation performance, but not with each other, fits neatly with our initial predictions: namely, that these two measures may each primarily recruit separate aspects of spatial representation (precision versus quantity) which are both utilized in mental rotation.

Our proposed explanation notwithstanding, one might suggest alternative explanations for an observed link between mental rotation and basic numerical skills. One possibility is that individuals who perform better at both skills simply exhibit better cognitive control. In this case, better mental rotation scores should predict less interference at numerical tasks that feature an automatically-processed irrelevant dimension which must be effortfully ignored (i.e. a smaller compatibility effect in the numerical comparison task). However, the relationship we observed between the MRS and the compatibility effect was exactly the opposite of this alternative prediction, thus suggesting that cognitive control was not the factor explaining this correlation. This is supported by findings from developmental studies which show that with age and experience (and better overall performance), the compatibility effect in children increases, suggesting that experience with numbers outweighs improved cognitive control (Mann et al., 2011; Nuerk, Kaufmann, Zoppoth, & Willmes, 2004). Additionally, no link was found between the MRS and measures of response randomness from the RNG task, which are considered to rely upon executive control (Brugger, 1997; Peters, Giesbrecht, Jelicic, & Merckelbach, 2007). Although the compatibility effect showed a significant correlation to randomness measures in the RNG task (see Table 1), this is not surprising, since both measures reflect some influence of executive function (Brugger, 1997; Nuerk & Willmes, 2005). In fact these findings render the cognitive control explanation even less plausible, by confirming that smaller compatibility effect was associated with better executive control (i.e. lower R or RNG index score, indicating better suppression of previous response selection). Together, these findings make it highly unlikely that cognitive control is the mediating factor underlying the connection in question. While it is not tenable to exclude the possibility that there are other mediating factors, the primary goal of the current study was to uncover the potential link between mental rotation abilities and basic numerical skills, and more specifically numerical representation.

It should be noted that a recent study by Macizo and Herrera (2011) compared mental rotation performance to the compatibility effect and found a relationship seemingly inconsistent with the results of the present study: namely, individuals in their experiment who exhibited a larger compatibility effect showed poorer mental rotation ability. However, the mental rotation task used by Macizo and Herrera (2011) differs fundamentally from the task we used (Peters et al., 1995) in several ways: type of rotation object (graphemes versus “3-dimensional” block objects), dimensions of rotation (vertical plane versus horizontal plane), response paradigm (forced-choice versus multiple choice), and psychometric measures (response time versus accuracy). (See Peters & Battista, 2008, for a further overview of the differences between these two tasks, and the consequent implications.) Given these extreme differences, it is possible that the two variations of the task capture minimally overlapping facets of mental rotation ability. Additionally, Macizo and Herrera (2011) measured the compatibility effect using response times (versus accuracy in our study) and analyzed the results of both compatibility effect and mental rotation by splitting scores into high versus low ability groups (rather than using correlation, as in our study). Such an approach (Extreme Group Approach) may affect findings in terms of many important factors, including reliability (Preacher, Rucker, MacCallum, & Nicewander, 2005). These discrepancies may partially account for the disparate findings between the two studies. However, given the various differences between the two studies (types, dimensions, measures), the determinants of the observed correlations should be examined further in future studies using different mental rotation tasks.

## 5 Conclusions and perspectives

From the present data it is clear that mental rotation and numerical skills are linked. This research opens new discussions in the field of developmental implications of the origin of this link between mental rotation and numerical representation; which precedes the other, or do they indeed develop simultaneously and in a connected fashion? Future studies that examine the developmental link between mental rotation abilities and numerical representation in young children, before and after formal education, might be able to assess the developmental trajectories of the mechanism that is proposed to underlie both skills. In fact, a recent study (Cheng & Mix, in press) has already shed some interesting light on this area: they showed that training children on a mental rotation task can improve later performance in mathematical tasks such as arithmetic. Although the study did not test basic numerical skills, it is altogether possible that this improvement was mediated by advances in number representation, and that spatial training could thus predict or improve other types of number skills. Future developmental studies may also be able to assess the likelihood of using mental rotation as one of the predictors for numerical competence before the acquisition of formal mathematical education. In contrast to predictors of numerical competence such as number line mapping tasks (Booth & Siegler, 2008), mental rotation is a task that requires no formal education, and can be performed with young children (Marmor, 1975) and even with infants, in the form of visual looking-time tasks with rotated versions of visually-habituated objects (Moore & Johnson, 2008; Quinn & Liben, 2008). If individual differences in mental rotation abilities at early developmental stages can partially predict later numerical abilities, this may be able to help corroborate other early warning signs of number difficulties. This in turn would allow maximum time for learning interventions in case of learning disabilities. However, such speculation at this stage calls for future studies that will deepen our understanding between mental rotation and numerical representation.

## Figures and Tables

**Table 1:** Values of correlation coefficients between measures from four tasks: Mental rotation, numerical comparison, number mapping, and RNG.

		Mental rotation score	Compatibility effect score	Number mapping accuracy	Small number bias	First order differences	Turning-point index	Redundancy score	RNG index	RNG2 index
Mental rotation score	r	1	.405⁎⁎	− .353⁎	.040	− .196	− .002	− .124	− .017	− .127
	p		.007	.020	.801	.207	.992	.435	.915	.422
Compatibility effect score	r		1	.065	.214	.031	− .233	− .308⁎	− .195	− .370⁎
	p			.677	.174	.842	.143	.047	.215	.016
Number mapping accuracy	r			1	− .093	− .131	− .191	− .150	.122	.015
	p				.558	.402	.232	.343	.441	.923
Small number bias	r				1	− .126	− .071	.214	− .273	.069
	p					.428	.659	.175	.080	.664
First order differences	r					1	.132	− .091	− .097	− .113
	p						.411	.567	.540	.475
Turning-point index	r						1	.120	.262	.126
	p							.455	.097	.433
Redundancy score	r							1	.267	.422⁎⁎
	p								.088	.005
RNG index	r								1	.108
	p									.495
RNG2 index	r									1
	p									

⁎ p < .05.

⁎⁎ p < .01.

## Footnotes

1 Due to well-documented sex differences in mental rotation and other number-related abilities (Geary et al., 2000; Johnson & Bouchard, 2007; Kucian, Loenneker, Dietrich, Martin, & Von Aster, 2005; Peters, Manning, & Reimers, 2007), we ran multiple regression analyses (using the enter method) to assess the fit of MRS and sex as predictors of both compatibility effect and number line mapping deviation. For both dependent variables, sex was not a significant predictor (compatibility effect: Beta = − .11, p = .44; number line mapping: Beta = − .19, p = .21), whereas MRS was a significant predictor (compatibility effect: Beta = .45, p < .01; number line mapping: Beta = − .38, p < .05), thus suggesting that the results of our observed correlations were not driven by sex differences.
